# Beyond accuracy: Cross-session stability in representation-based animal identification

**DOI:** 10.1016/j.isci.2026.117004

**Published:** 2026-08-03

**Authors:** Alvaro Rodriguez, Ehsan Noshahri, Alejandro Puente-Castro, Andres Molares-Ulloa

**Affiliations:** 1 Universidade da Coruña, Department of Computer Science and Information Technology, 15071 A Coruña, Spain; 2 Centro de investigación CITIC, 15071 A Coruña, Spain

**Keywords:** animal identification, identity consistency, representation learning, multi-session experiments, animal studies

## Abstract

Identification of unmarked animals is central to behavioral and ecological research. Representation learning currently achieves near-perfect performance in single-session experiments. However, stability across sessions remains poorly characterized. We evaluate identity consistency, accuracy, and learned representation using synthetic and real cross-session experiments. The results show that identification degrades even under minimal visual changes. Although exposure to multiple sessions improves generalization, substantial degradation remains. This indicates that the reported accuracy alone is insufficient to guarantee stable identities across sessions.

## Highlights

• Identity consistency degrades under cross-session evaluation
• Minimal visual changes disrupt learned identity representations
• Learned representations remain dependent on recording context
• Within-session accuracy does not guarantee longitudinal identity stability

## Graphical abstract

**Figure:**
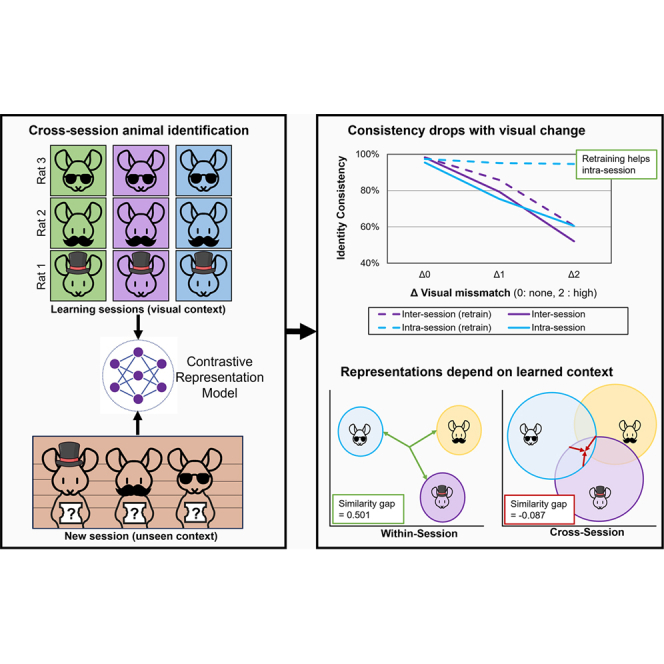

## Teaser

Biological sciences; machine learning; zoology

## Introduction

Understanding animal behavior often requires the reliable identification of individuals over time. In ecological and behavioral research, individual-level information is essential to quantify social structure, movement, and behavioral dynamics. Traditionally, animal identification has relied on physical marking or manual annotation, approaches that are time consuming and difficult to scale and may interfere with natural behavior. As experimental datasets increase in size and complexity, the need for automated, non-invasive methods capable of identifying unmarked individuals continues to grow.

Early efforts to automate identification combined computer vision-based tracking with appearance cues to distinguish unmarked animals. Systems such as idTracker^1^ and ToxTrac^2^^,^^3^ represent important milestones, enabling the automated tracking and identification of multiple individuals under controlled conditions. These approaches demonstrated that identity preservation was feasible using visual information alone. However, their reliance on hand-crafted features and probabilistic texture analysis limited their applicability to short experiments involving small numbers of animals.^4^

The introduction of deep learning^5^ improved accuracy and scalability to larger groups by learning discriminative features directly from the data. However, these gains came at a computational cost, with training and inference times ranging from hours to weeks, limiting their practical use.

To address these limitations, recent work has reframed animal identification as a representation learning problem. In this paradigm, models are trained to map images into a discriminative feature space where individuals can be separated.^6^ Within this framework, contrastive learning has enabled faster training and improved scalability by leveraging pairwise comparisons.^7^

This shift has led to a new generation of methods, which we will refer to as contrastive representation pipelines (CRPs) that reduce processing times while maintaining strong identification performance, as exemplified by recent multi-object tracking systems.^8^^,^^9^ Among these, “new idtracker.ai”^10^ represents a notable milestone, reporting identification accuracies close to 99.9% for groups of up to 100 unmarked individuals under controlled conditions.

CRPs are increasingly being used in longitudinal studies, with mechanisms that propagate identity labels and representations across sessions, known, respectively, as identity transfer and transfer learning. However, these mechanisms have not been systematically evaluated. More broadly, their reliability depends on whether information learned in one session remains valid under new conditions, a problem known as generalization and recognized as an important challenge in machine learning and representation learning.^11^^,^^12^

Furthermore, recent studies in wildlife re-identification,^13^ dairy cow recognition,^14^ identification of Amur tigers,^15^ and others have reported accuracies typically ranging between 83% and 95%, well below the near-perfect performance reported in controlled single-session tracking settings. These results suggest that reported identification performance does not necessarily translate into stable identity preservation under new conditions, potentially affecting downstream analyses that rely on individual-level continuity, including estimates of trajectories, interaction patterns, or social structure.

We examine whether identity assignments by CRPs remain stable across recording sessions. We address this question using controlled visual perturbations and a multiple-session experiment designed to assess identity generalization under natural session-to-session variability.

## Results

### Visual perturbation results

#### Identity consistency across sessions

**Figure 1:**
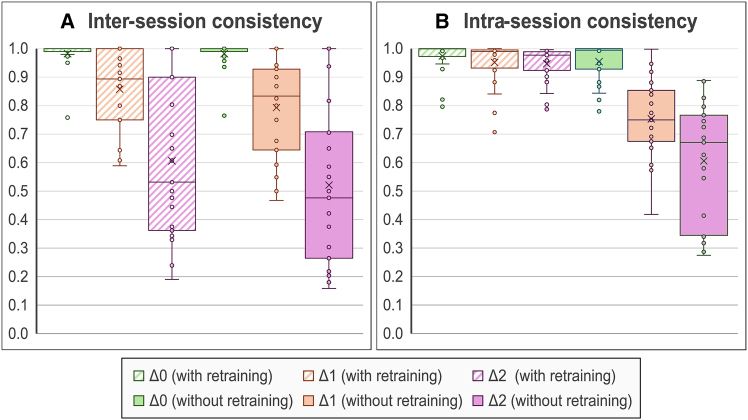
Identity consistency across sessions and visual perturbation levels (A and B) Inter-session consistency (A) and intra-session consistency (B) across three levels of visual mismatch (Δ0, Δ1, and Δ2), evaluated with and without retraining. Each boxplot summarizes consistency values across all datasets and species. Boxes indicate interquartile ranges, horizontal lines denote medians, crosses indicate means, and points correspond to dataset measurements. Consistency decreases as Δ increases, with retraining partially mitigating degradation, particularly for the intra-session condition.

**Figure 2:**
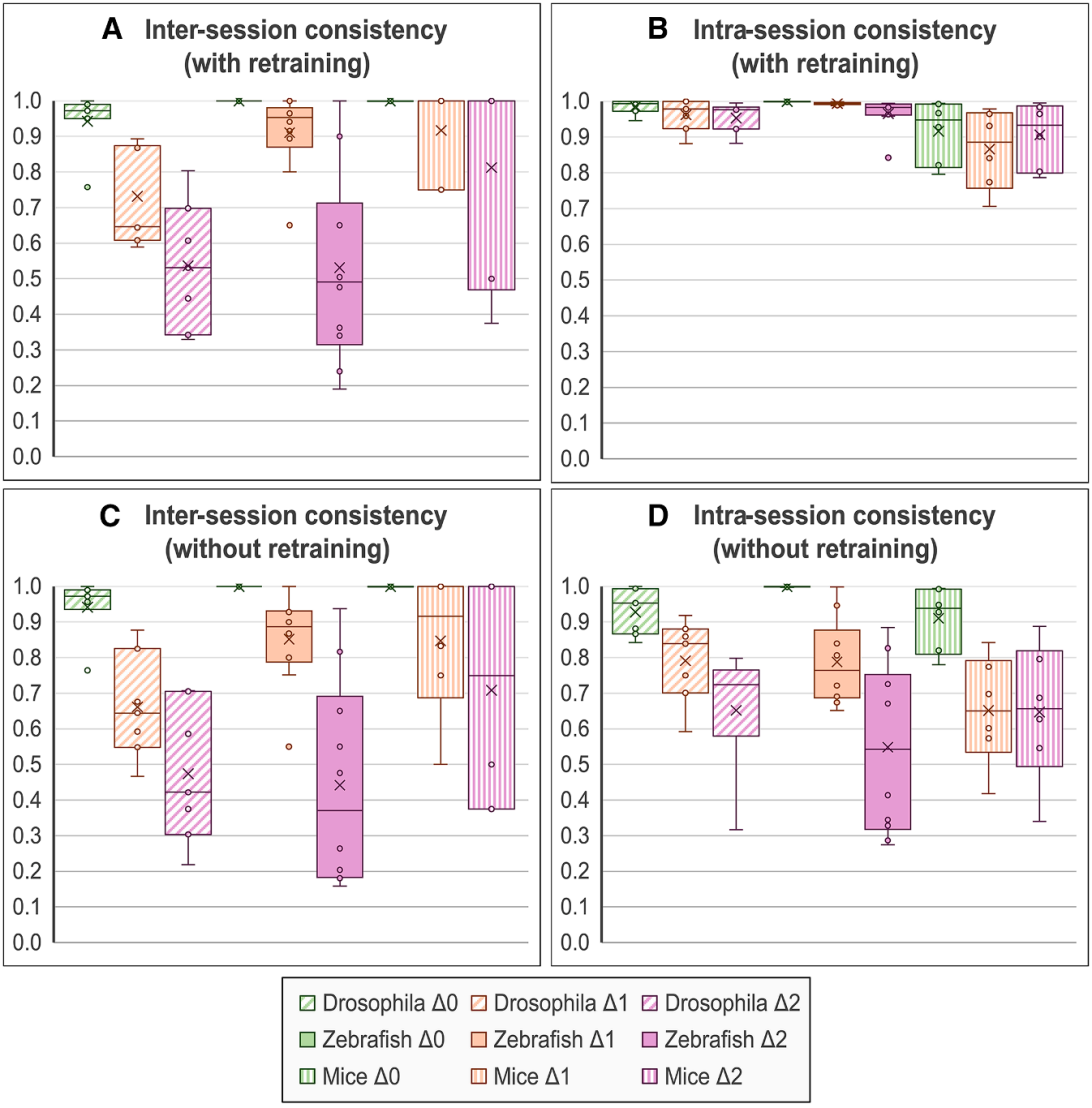
Species-specific identity consistency across visual perturbation levels (A–D) Inter-session (A and C) and intra-session (B and D) identity consistency for *Drosophila*, mice, and zebrafish, evaluated with retraining (A and B) and without retraining (C and D), across three levels of visual mismatch (Δ0, Δ1, and Δ2). Boxplots summarize dataset-level consistency values for each species at Δ0, Δ1, and Δ2. While baseline consistency differs across species and group sizes, all species exhibit systematic degradation as Δ increases, with retraining improving robustness but not fully preventing consistency loss.

Identity consistency quantifies whether identity assignments remain preserved between analyses under different conditions (see STAR Methods, Figures 1 and 2).

**Figure 3:**
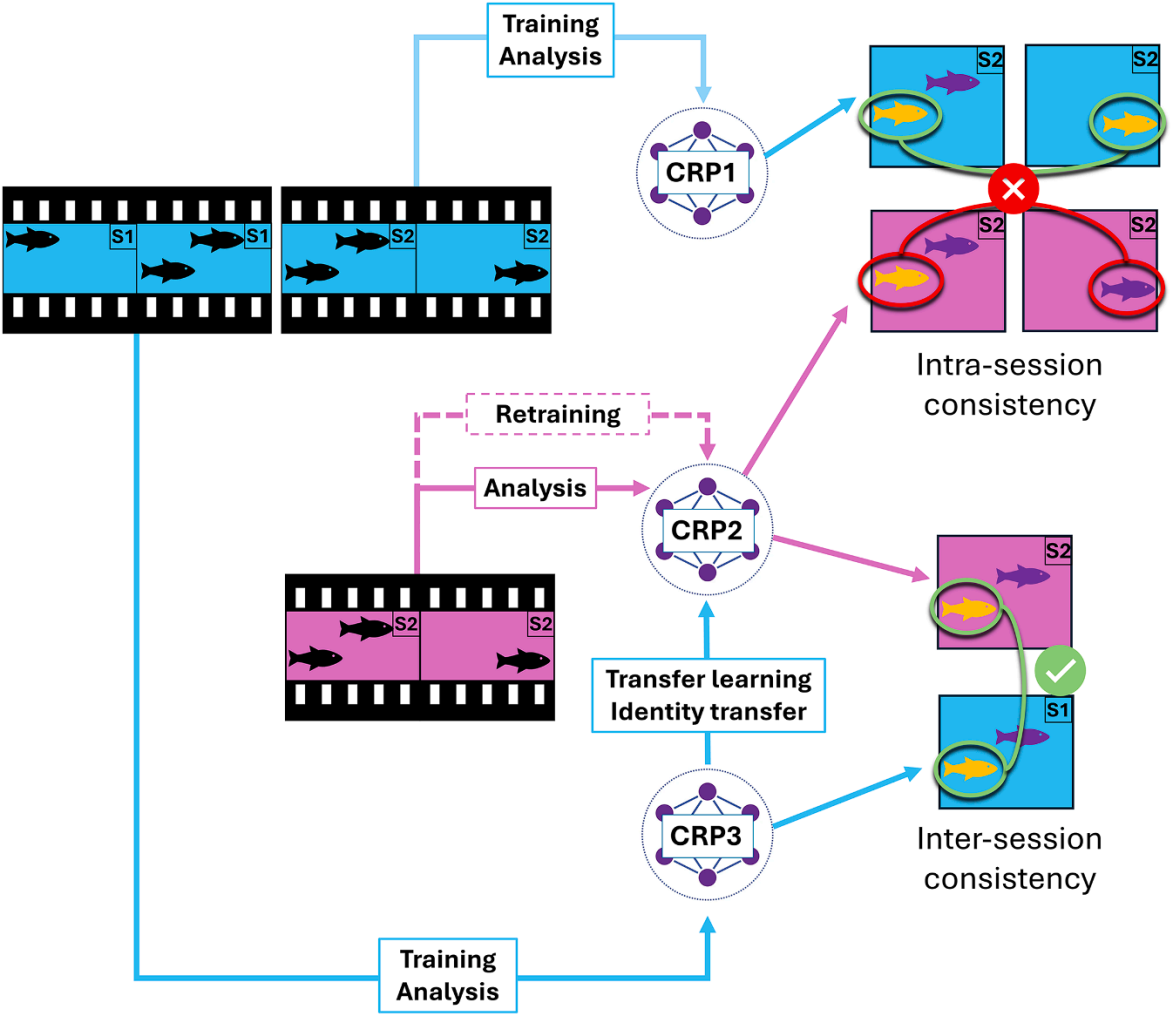
Experimental design Each contrastive representation pipeline (CRP) denotes a full identification workflow, including representation learning and identity assignment. Session labels (S1 and S2) indicate temporal segmentation of the same recording. Color encodes the level of visual perturbation. (i.e., blue corresponds to visual perturbation level 0, and pink to level 2, creating a Δ2 visual mismatch). CRP1 is trained and applied on session 1, CRP3 is trained and applied on session 2 under unperturbed conditions, and CRP2 is initialized from CRP1 and applied to session 2 with identity transfer, transfer learning, and optional retraining. Inter-session consistency is evaluated by comparing CRP1 and CRP2 at the anchor frame, while intra-session consistency compares CRP2 and CRP3 within session 2.

Figure 3 summarizes identity consistency across all datasets for inter- and intra-session evaluations, with and without retraining as a function of increasing levels of visual mismatch across sessions Δ0, Δ1, and Δ2.

Under Δ0, identity consistency is high for both inter- and intra-session evaluations. Notably, identity consistency is not perfect even at Δ0, indicating that identity instabilities may arise even under the most favorable conditions. The lowest inter-session Δ0 value corresponded to a single dataset with animal crossings in the anchor frame used to estimate the metric, likely explaining the reduced baseline consistency. Likewise, a small number of datasets exhibited reduced intra-session consistency. In most of these cases, retraining restored consistency to near-perfect levels, indicating that the reductions were caused by the learned representations rather than by the datasets themselves. The remaining cases corresponded to datasets containing only two individuals, where small discrepancies have a comparatively large effect on consistency.

Inter-session consistency evaluation (Figure 1A) shows that without retraining, consistency decreases sharply as Δ increases. Retraining provides only marginal improvement.

Intra-session consistency evaluation (Figure 1B) shows that without retraining, consistency degrades sharply as Δ increases. With retraining, intra-session consistency shows only a modest decrease.

These results demonstrate that identity consistency is strongly dependent on the similarity between sessions and that retraining alone does not guarantee stable identity preservation under moderate-to-strong visual changes.

#### Species and group size effects

**Figure 4:**
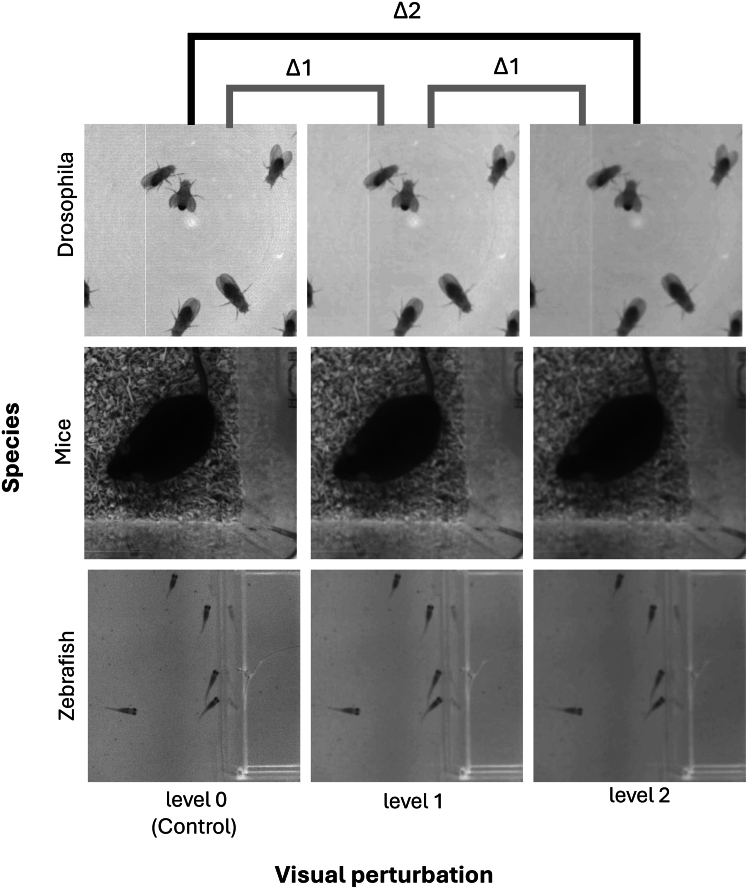
Example of visual perturbations and variation across sessions Example 300 × 300 pixels enlarged crops from *Drosophila*, mice, and zebrafish under three visual perturbation levels. Level 0 corresponds to the control condition. Level 1 applies a 5% brightness increase, Gaussian blur (σ = 1), and contrast reduced to 93%. Level 2 applies a 5% brightness increase, Gaussian blur (σ = 1.5), and contrast reduced to 87%. Lines illustrate Δ-levels: Δ1 corresponds to a one-level mismatch and Δ2 to a two-level mismatch, as used in the experimental comparisons.

Figure 4 shows identity consistency stratified by species. While overall trends are consistent across species, the magnitude of degradation varies. Detailed results for individual datasets and conditions are provided in Tables S3–S6.

Identity consistency degradation is less pronounced in mice and more pronounced in datasets involving denser scenarios. Zebrafish and *Drosophila* recordings, which include larger group sizes of smaller and more visually uniform animals, show a faster decline in both inter-session and intra-session consistency compared with those in mice, and retraining provides only partial recovery.

Overall, these results indicate that while the effect of Δ is consistent across species, the magnitude of consistency depends on group size and the appearance of the species.

#### Summary statistics

Table 1 reports the mean and minimum identity consistency for each Δ-level, and Table 2 reports degradation with visual mismatch. Mean values follow the trends observed in the boxplots, with consistent decreasing from Δ0 to Δ2 and systematic improvements with retraining. Minimum values reveal worst-case behavior. Notably, even under Δ0 conditions, minimum consistency values fall below perfect consistency for both inter-session and intra-session evaluations. Under Δ2, minimum values drop sharply, particularly without retraining, highlighting substantial variability and potential failure cases.

**Table 1:** Summary of results

Metric	Retraining	Δ0 mean	Δ1 mean	Δ2 mean	Δ0 min.	Δ1 min.	Δ2 min.
Inter-session consistency	yes	0.98	0.86	0.61	0.76	0.59	0.19
Inter-session consistency	no	0.98	0.79	0.52	0.76	0.47	0.16
Intra-session consistency	yes	0.97	0.95	0.95	0.80	0.71	0.79
Intra-session consistency	no	0.95	0.75	0.61	0.78	0.42	0.27

Mean and minimum identity consistency for three levels of visual mismatch (Δ0, Δ1, and Δ2) reported separately for inter-session and intra-session evaluations, with and without retraining.

**Table 2:** Relative degradation with increasing visual mismatch

Retraining	Δ0→Δ1	Δ0→Δ2
Yes (inter-session)	−12.2%	−37.8%
No (inter-session)	−19.4%	−46.9%
Yes (intra-session)	−2.1%	−2.1%
No (intra-session)	−21.1%	−35.8%

Percentage reduction in mean identity consistency relative to the corresponding Δ0 condition for each evaluation type and retraining strategy. Values indicate the proportional loss of consistency observed at Δ1 and Δ2 with respect to the baseline condition. Larger negative values correspond to stronger degradation of identity consistency.

These results emphasize that identity consistency cannot be assumed to be uniformly reliable, even when the average performance appears high.

#### Mixed-effects analysis

To evaluate the effects of visual perturbation while accounting for repeated measurements across datasets, we fitted a linear mixed-effects model using identity consistency as the response variable. We included the perturbation level (Δ), consistency type (inter-session or intra-session), retraining condition, species, and group size as fixed effects and the dataset as a random intercept.

The mixed-effects model confirmed that visual perturbation was the strongest predictor of consistency degradation (Table 3). Both Δ1 and Δ2 significantly reduced identity consistency, whereas retraining improved consistency, larger group sizes were associated with lower consistency, and species had no significant effect. The model also revealed that the effect of strong perturbations differed significantly between the evaluation types, with substantially larger reductions for inter-session consistency than for intra-session consistency under Δ2 perturbations. Separate analyses for each consistency type yielded similar conclusions (Tables S7 and S8).

**Table 3:** Summary of mixed-effects model

Predictor	Effect	β, beta (effect size)	95% CIs	*p* value
Δ1	reduced consistency	−0.157	[-0.220, −0.095]	<0.001
Δ2	reduced consistency	−0.418	[-0.481, −0.356]	<0.001
Retraining	improved consistency	+0.118	[0.081, 0.154]	<0.001
Group size	reduced consistency with larger groups	−0.001	[-0.002, −0.000]	0.003
Species (mice)	not significant	−0.032	[–0.126, 0.063]	0.509
Species (zebrafish)	not significant	+0.013	[-0.055, 0.081]	0.706
Δ2 × consistency type	larger Δ2 effect in inter-session evaluations	0.23	[0.142, 0.319]	<0.001

Results of the linear mixed-effects model fitted using identity consistency as the response variable. Perturbation level (Δ), retraining condition, consistency type (inter-session or intra-session), species, and group size (number of animals) were included as fixed effects, and dataset was included as a random intercept. The table reports the estimated regression coefficients (β), 95% confidence intervals (CIs), and *p* values for the effects included in the model. Coefficients are expressed relative to the baseline condition (inter-session consistency, Δ0, no retraining, and *Drosophila*).

Together, these results confirmed that the observed degradation is primarily associated with increasing visual mismatch and that this effect is particularly pronounced in the inter-session condition.

### Multiple-session results

**Figure 5:**
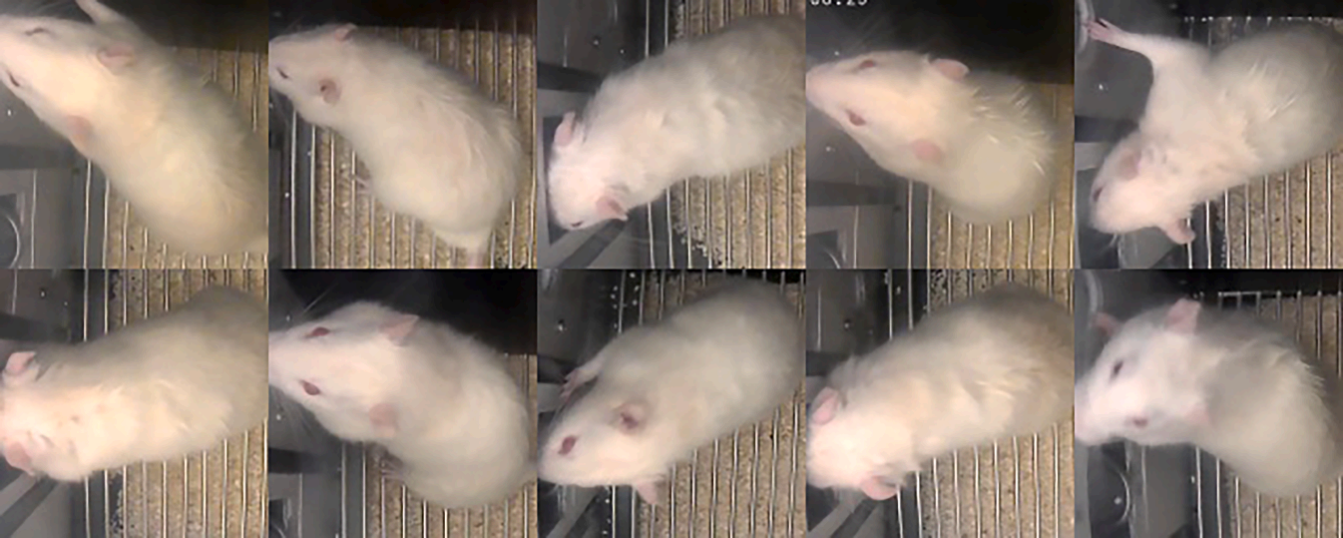
Representative images from the multiple-session dataset Example images for the ten rats used in the experiment. Images were acquired in operant conditioning chambers during different recording sessions and illustrate appearance variability encountered in longitudinal designs. Image resolution: 128 × 128 pixels

To evaluate identity generalization, we compared within-session and cross-session evaluation settings using models trained with either single-session or multi-session data (see STAR Methods, Table 4, Figure 5). Table 5 summarizes identification performance measured by Top-1 accuracy and representation structure quantified by the similarity gap, where positive values indicate that samples are closer to their correct identities than to the competing ones, whereas negative values indicate loss of identity separation.

**Table 4:** Summary of evaluation protocol

Model	Training	Test
Single-session	single session	same session
Single cross-session	single session	different session
Multi-session	multiple sessions	seen sessions
Multi cross-session	multiple sessions	unseen sessions

Training and test partitions used a holdout protocol with a 70/30 split for all models. We reserved 20% of the training data for validation. To avoid data leakage, we defined session partitions before image randomization. Reported results correspond to the mean and standard deviation across nine independent train-test configurations for each evaluation setting (see STAR Methods for additional details).

**Table 5:** Summary of results

Model	Top-1 accuracy	Mean positive similarity	Mean best negative similarity	Similarity gap
Single-session	0.951 ± 0.026	0.884 ± 0.023	0.299 ± 0.037	0.585 ± 0.052
Single cross-session	0.169 ± 0.061	0.125 ± 0.077	0.577 ± 0.051	−0.452 ± 0.081
Multi-session	0.928 ± 0.019	0.807 ± 0.023	0.306 ± 0.025	0.501 ± 0.045
Multi cross-session	0.437 ± 0.060	0.406 ± 0.065	0.494 ± 0.023	−0.087 ± 0.071

Identification performance and embedding-space structure for the four evaluation settings. “Single-session” and “single cross-session” models were trained using data from a single session, whereas “multi-session” and “multi cross-session” models were trained using multiple sessions. Performance is reported as mean ± standard deviation across the evaluated session combinations.

Both the “single-session” and “multi-session” models achieved high identification accuracy (>0.9) and large positive similarity gaps.

In contrast, cross-session evaluation produced a marked reduction in performance. The “single cross-session” model showed poor transfer of identity representations, reflected by a strongly negative similarity gap.

Training with multiple sessions substantially improved cross-session performance and reduced the magnitude of the negative similarity gap. However, performance remained markedly below within-session levels and the similarity gap remained negative.

Together, these results indicate that exposure to multiple sessions improves robustness to session-to-session variability but does not produce fully session-invariant identity representations.

Paired statistical comparisons across the nine rotating session configurations confirmed the observed reductions in accuracy and similarity gap under cross-session evaluation for both single-session and multi-session training schemes (Table S9). All comparisons were significant (*p* < 0.001), with large effect sizes and 95% confidence intervals excluding zero.

## Discussion

Metrics commonly reported in the literature, typically derived from within-session evaluations, provide little guidance on how stable identities remain when trained systems are applied to new sessions.

The mixed-effects analysis confirmed that visual perturbation is the strongest predictor of consistency degradation, while retraining partially mitigates this effect. Furthermore, consistency decreased significantly with the increasing group size, whereas species itself was not a significant predictor after accounting for the other variables. Most importantly, the effect of perturbation was significantly stronger for inter-session consistency than for intra-session consistency. Separate mixed-effects analyses showed that retraining strongly improved intra-session consistency but had only a limited effect on inter-session consistency (Tables S7 and S8). These results reveal a substantial gap between the high within-session performance commonly reported in the literature and the level of identity stability required for reliable longitudinal studies.

The multiple-session experiment supports and extends this interpretation. When training and evaluation were performed under the same conditions, identification accuracy remained high and identity representations were well separated. In contrast, cross-session evaluation led to a marked performance reduction and negative similarity gaps, indicating that samples were no longer closer to their correct identity prototypes than to competing identities. Training with multiple sessions substantially improved cross-session performance, showing that the exposure to session variability can partially mitigate the problem. However, performance remained far below within-session levels, and the similarity gap remained negative, with statistical comparisons confirming large and significant differences relative to within-session evaluations (Table S9).

Together, these findings suggest that current representation-based identification pipelines adapt primarily to the visual characteristics present during training. Retraining improves consistency by updating representations to the context of a new session, while training with multiple sessions improves robustness by incorporating a broader range of visual variability from the start. However, neither strategy fully eliminates performance degradation under new conditions. This suggests that the learned identity features remain strongly dependent on the recording context and do not remain stable across sessions.

This work examined the stability of animal identity assignments across sessions, using both controlled visual perturbations and real multi-session recordings. Using CRPs implemented in “new idtracker.ai” and supervised embedding-based identification models, we evaluated whether identity representations learned under one set of recording conditions remain stable when applied to new sessions.

Identity assignments across sessions were not reliable, even when session differences were minimal. Although retraining and training with multiple sessions substantially improved cross-session performance, substantial degradation remained.

These findings indicate that current representation-based identification systems learn part of the variability associated with different recording conditions but do not achieve fully session-invariant identity representations.

Consequently, the reliable application of these systems to multiple-session studies requires strategies that address session variability beyond those used in experimental practice today. Possible directions include expanding the use of controlled visual perturbations and data from multiple sessions during training or modeling the transformation between identity representations across sessions.

For researchers, this implies that using representation-based systems as black-box identity providers is not warranted unless identity consistency is explicitly assessed. Maintaining stable image conditions may reduce the risk of systematic errors, but identity assignments should still be validated whenever possible. More broadly, maintaining identity consistency remains an open challenge.

### Limitations of the study

This study has three main limitations. First, the controlled visual perturbation experiments isolate the effect of session-to-session visual variability but do not capture all sources of variation present in real longitudinal studies. Although the perturbations were designed to represent realistic changes in recording conditions, other factors may also influence identity stability.

Second, the multiple-session evaluation was conducted using a single longitudinal dataset. While this experiment complements the controlled perturbation analysis by demonstrating similar behavior under natural recording variability, additional datasets covering different species, acquisition protocols, and experimental conditions would be valuable to assess the generality of these findings.

Finally, our conclusions are based on representative CRPs and supervised embedding-based identification models. The observed limitations, therefore, characterize current approaches evaluated in this study and should not be interpreted as evidence that session-invariant identity representations are fundamentally unattainable.

## Resource availability

### Lead contact

Requests for further information and resources should be directed to and will be fulfilled by the lead contact, Alvaro Rodriguez (a.tajes@udc.es).

### Materials availability

This study did not generate new unique materials.

### Data and code availability

• All datasets used in this study are publicly available and can be accessed via the links provided in the key resources table.
• All custom code is available on request from the lead contact.
• Any additional information required to reanalyze the data reported in this paper is available from the lead contact upon request.

## Acknowledgments

This work was partially funded by the EU through the FEDER Galicia 2021-27 operational program ref. ED431G 2023/01; by the Consellería de Educación, Ciencia, Universidades e Formación Profesional of Xunta de Galicia under the program “Axudas para a consolidación e estruturación de unidades de investigación competitivas (Grupos de Referencia Competitiva)”, ref. ED431C 2026/56; by grant PID2021-126289OA-I00 funded by 10.13039/501100004837MCIN/10.13039/501100011033AEI/https://doi.org/10.13039/501100011033 and 10.13039/501100008530ERDF A way of making Europe; and by the 10.13039/501100007601European Union’s Horizon 2020 research and innovation program under the Marie Skłodowska-Curie grant agreement no. 101034261.

## Author contributions

A.R. defined the experimental design, wrote the manuscript, created the figures, wrote the code, and collaborated in all the experiments and data analysis; E.N. wrote the manuscript and collaborated in the visual perturbation experiments and data analysis; A.M.-U. revised the manuscript, created the figures, wrote the code, and collaborated in the multiple-session experiment and data analysis. A.P.-C. revised the manuscript and collaborated in the statistical models and data analysis.

## Declaration of interests

The authors declare no competing interests.

## Declaration of generative AI and AI-assisted technologies in the writing process

The authors used ChatGPT to improve language and grammar. The authors reviewed and edited the article and take full responsibility for its content.

## STAR★Methods

### Key resources table

**Table:** 

REAGENT or RESOURCE	SOURCE	IDENTIFIER
**Deposited data**		

Rat ID dataset	Universidade da Coruña, Department of Computer Science and Information Technology, Spain	Zenodo Data: https://doi.org/10.5281/zenodo.15112879
Visual perturbation dataset	Champalimaud Research, Champalimaud Center for the Unknown, Portugal	https://idtracker.ai/

**Software and algorithms**		

*new idtracker.ai* (v6.0.9)	Champalimaud Research, Champalimaud Center for the Unknown, Portugal	http://idtracker.ai/
VirtualDub2	Anton Shekhovtsov	http://virtualdub2.com/

### Method details

We used two complementary experimental designs to evaluate whether identity representations remain stable under changing recording conditions.

First, we evaluated identity consistency in contrastive representation pipelines (CRPs) implemented in *new idtracker.ai* using controlled visual perturbations. Second, we evaluated identification performance and representation structure across multiple sessions using supervised embedding-based identification models.

#### Visual perturbation pipeline

We use *new idtracker.ai*^10^ as a representative of CRPs to quantify identity consistency within and across recording sessions (Figure 3).

CRPs rely on deep learning models that map animal images into a representation space capturing individual visual differences. Training is driven by contrastive learning, which exploits two structural assumptions: animals detected simultaneously in the same frame correspond to different individuals, whereas temporally contiguous detections of an isolated animal correspond to the same individual. These assumptions allow the construction of pairs of images of different animals (negative pairs) and of the same animal (positive pairs), respectively.

During training, the model pulls representations of positive pairs closer while pushing representations of negative pairs farther apart in the representation space.^16^ The resulting representation separates individuals based on visual similarity learned from the data.^6^ Identity assignments are then performed by comparing new images to the learned identity clusters.^17^

In a cross-session setting, learned identity labels and representations are propagated to a new session through identity transfer and transfer learning, respectively. When retraining is enabled, the transferred representations are further updated using data from the new session through fine-tuning.

#### Visual perturbation datasets and conditions

Experiments were conducted on video recordings of *Drosophila* flies, mice and zebrafish, covering a wide range of group sizes and appearances. All videos were selected from the datasets released with *new idtracker.ai*^10^ following a controlled population-based criterion.

To obtain a balanced and scalable experimental corpus, videos were selected until reaching 500 individuals per species group. This included all available mice recordings, and zebrafish and *Drosophila* recordings until the target population was reached.

As the original recordings varied in duration, all videos were temporally standardized by using the first 4 min of each video.

Dataset characteristics, including species, number of individuals, duration, and imaging setup, are summarized in Table S1. To evaluate whether the applied perturbations produced substantial changes in segmentation quality, additional dataset-level segmentation diagnostics, including segmentation rate, crossing frequency, and trajectory fragmentation, are reported in Table S2.

To evaluate identity consistency under controlled variability, we introduce perturbation levels applied to the input videos prior to identification. We define three experimental conditions (Figure 4). Under Δ0, Session 1 and Session 2 are used without perturbation. Δ1 and Δ2 introduce moderate and stronger perturbations of blur, brightness, and contrast, defining increasing mismatch between sessions.

For each Δ-level, all perturbation combinations consistent with that mismatch are evaluated and averaged to obtain a single identity consistency estimate.

#### Visual perturbation reproducibility details

For the visual perturbation design, both reference CRPs and retrained CRPs used *new idtracker.ai* (v6.0.9). CRPs operated without retraining used a modified version of new idtracker.ai (v6.0.9), in which model parameters were kept fixed after training on Session 1 and no parameter updates were performed when processing Session 2.

To avoid variations caused by small segmentation differences, we used a threshold of five pixels for *Drosophila* and zebrafish and ten pixels for mice when estimating identity consistency from animal positions.

The visual perturbation datasets were used with the processing parameters published by the authors when possible (area threshold was increased for mice from recommended to 100,000 pixels to avoid filtering out valid detections).

VirtualDub2 was used for video editing and generation of the visual perturbation levels. Videos were processed using the following sequence: (1) Gaussian blur, (2) brightness/contrast adjustment, and (3) video export. All videos were re-encoded using the x264 8-bit H.264/MPEG-4 AVC codec with a single-pass Constant Rate Factor (CRF) of 15.0. For Δ1, the built-in Gaussian blur filter was applied with a radius of 1 pixel, followed by the built-in brightness/contrast filter with +5% brightness and 93% contrast. For Δ2, the built-in Gaussian blur filter was applied with a radius of 1.5 pixels, followed by the built-in brightness/contrast filter with +5% brightness and 87% contrast.

#### Multiple session pipeline

To independently evaluate identity generalization under natural session-to-session variability, we trained a ResNet50 contrastive identification model. Similar to the CRPs described above, the model was trained to produce embeddings that maximize similarity between images of the same individual while increasing separation between different individuals.

#### Multiple session dataset and conditions

The multiple session design used the Rat ID dataset.^18^ Representative images from the dataset are shown in Figure 5. The experimental conditions of this dataset consist of repeated recordings of the same individuals acquired across multiple sessions in the same environment but on different days, preserving natural variation in acquisition conditions, including changes in camera position, illumination, focus, and field of view. For this experiment, we selected 10 rats recorded across nine sessions. Full dataset details are available in the original publication.

#### Multiple session reproducibility details

Models in the multiple session experiments were trained using the same supervised contrastive learning pipeline based on a ResNet50 backbone.^19^ Identity embeddings had a dimensionality of 256 and were optimized using a projection head of 128 units. Training employed a batch size of 256, a contrastive temperature of 0.07, and the Adam optimizer with a learning rate of 0.001.

We used a holdout cross-validation strategy with a 70/30 train-test split and 20% of the training data reserved for validation. Each session was split before randomization to prevent data leakage between training, validation, and test sets. To ensure comparability across conditions, all experiments used fixed dataset sizes of 28,000 training images, 7,000 validation images, and 15,000 test images.

Single-session model experiments were repeated across nine train-test configurations, corresponding either to within-session or cross-session evaluations. Multi-session model experiments were also repeated across nine rotating train-test configurations, using five sessions for training and either the same five sessions or the remaining four sessions for testing. Reported results correspond to the mean and standard deviation across the nine repetitions of each condition.

Early stopping was applied independently to the two training stages (patience of 5 and 3 epochs, respectively). The maximum number of epochs was set to 50 for the contrastive training stage and 20 for the second optimization stage. All experiments used identical hyperparameters and training procedures.

### Quantification and statistical analysis

The following procedures describe how identity consistency, identification performance and embedding-space structure were quantified throughout the study.

#### Visual perturbation evaluation protocol

To obtain an optimistic and internally consistent baseline aligned with the design assumptions of *new idtracker.ai*, each recording is divided into two consecutive sessions (Session 1 and Session 2). Both sessions originate from the same video and share identical acquisition conditions, experimental setup, and animal composition. Each session spans 2 min of video.

The two sessions overlap by a single frame, which provides an unambiguous correspondence between individuals and serves as an anchor for evaluation. Δ0 conditions represent the most favorable scenario for cross-session generalization. The visual perturbation experiments introduce progressively stronger visual mismatches, allowing the effect to be isolated and quantified.

Inter-session identity consistency is evaluated by comparing identity assignments produced by a system trained and applied to Session 1 under unperturbed conditions with those produced by a system initialized using Session 1 and applied to Session 2, with and without retraining, under increasing Δ-levels.

Intra-session identity consistency is evaluated by comparing identity relations produced within Session 2 by the transferred system with those produced by a reference system trained and applied to Session 2 under unperturbed conditions.

#### Visual perturbation session validation

As an additional validation, we verified that the session-construction procedure did not alter the baseline performance. The datasets analyzed in this study correspond to recordings evaluated with *new idtracker.ai*, reporting accuracies >99%.^10^ To confirm that splitting and re-encoding videos did not introduce artifacts, we applied the standard *new idtracker.ai* identification procedure to the unperturbed Δ0 sessions and compared the resulting identities with those obtained from the corresponding original recordings (without using identity transfer or transfer learning). Agreement (unique individuals map to the same positions without switches) exceeded 99%, confirming that baseline identity assignments were preserved.

#### Visual perturbation metrics

Identity consistency is quantified as the proportion of preserved identities or identity relations under each experimental condition. Identity consistency should not be interpreted as recognition accuracy. Recognition accuracy measures agreement between predicted identities and independently established ground-truth identities. In contrast, identity consistency measures the degree to which identity assignments remain preserved between different analyses, sessions, or experimental conditions.

**Inter-session consistency** measures generalization. It is the proportion of individuals whose identity in Session 1 remains preserved in Session 2 after transferring identities and representations learned in Session 1 to Session 2. It is estimated using an overlapping frame between both sessions as a reference to compare identity assignments.

**Intra-session consistency** measures identity stability within Session 2. It is the proportion of identity relationships that remain preserved after transferring identities and representations learned in Session 1 to Session 2. This metric is estimated from 500 randomly sampled non-repeating observation pairs per individual within Session 2. Pairs of observations assigned to an individual by the reference system are considered consistent if both observations remain grouped under one identity after transfer.

Identity consistency values are reported for each Δ-level (Δ0, Δ1, Δ2), and for both retraining conditions.

#### Multiple session evaluation protocol

We created four complementary evaluation settings (Table 5). The *Single-session* and *Single cross-session* models evaluate identification performance when training is restricted to a single session, while the *Multi-session* and *Multi cross-session* models assess the effect of exposure to multiple sessions during training. Together, these settings provide an evaluation of identity generalization under natural session-to-session variability.

#### Multiple session metrics

We used classification accuracy and prototype proximity (the cosine of the angle between sample embeddings and identity prototypes in the embedding space) to evaluate identification performance and embedding-space structure. In particular, we used:

**Top-1 accuracy** is the proportion of samples assigned to their correct identity prototype.

**Mean positive similarity** is the average proximity between each sample embedding and the prototype corresponding to its true identity. Higher values indicate that samples remain closely associated with their expected identity representation.

**Mean best negative similarity** is the average proximity between each sample embedding and the most similar competing identity prototype. Higher values indicate greater overlap between identity representations.

**Similarity gap** is the difference between mean positive similarity and mean best negative similarity. Positive similarity gaps indicate that samples remain closer to their correct identity representation than to competing identities, whereas negative similarity gaps indicate a loss of identity separation in the embedding space.
