# Supplementary material for: Beyond accuracy: Cross-session stability in representation-based animal identification
Source: iScience. 2026 Aug 3;29(8):117004. doi: 10.1016/j.isci.2026.117004 (PMC13469807; doi:10.1016/j.isci.2026.117004)
Supplement: Supplementary file 1 [file mmc1.pdf]

iScience, Volume *29*

Supplemental information

Beyond accuracy: Cross-session stability in representation-based animal identification

Alvaro Rodriguez, Ehsan Noshahri, Alejandro Puente-Castro, and Andres Molares-Ulloa

Beyond accuracy: cross-session stability in representation-based animal identification

Alvaro Rodriguez [a,b,*], Ehsan Noshahri[a,b], Alejandro Puente-Castro[a,b]
Andres Molares-Ulloa[a,b]

[a] *Universidade da Coruña, Department of Computer Science and Information Technology, 15071, A Coruña, Spain*

[b] *Centro de investigación CITIC, 15071, A Coruña, Spain.*

[*] *Corresponding author: a.tajes@udc.es (Alvaro Rodriguez).*

Supplementary Tables

Table S1: Dataset information. For each recording, the table reports the original video name, number of individuals, spatial resolution, frame rate (fps), session duration (in frames), total duration (in frames and seconds), and imaging setup. All videos were selected from the datasets released with *new idtracker.ai*.

Dataset	Group size	Resolution X	Resolution Y	fps	Frames Session	Frames Total	Seconds Total
drosophila_010	10	1280	960	60.00	7200	14400	240
drosophila_038	38	3456	3400	36.00	4320	8640	240
drosophila_072	72	3456	3400	36.00	4320	8640	240
drosophila_080	80	3712	3600	34.00	4080	8160	240
drosophila_100_1	100	3712	3712	28.00	3360	6720	240
drosophila_100_2	100	2432	2400	51.00	6120	12240	240
drosophila_100_3	100	3712	3600	34.00	4080	8160	240
mice_2_1	2	984	556	49.17	5900	11800	240
mice_2_2	2	984	556	49.17	5900	11800	240
mice_2_3	2	984	556	49.17	5900	11800	240
mice_2_4	2	984	556	49.17	5900	11800	240
mice_4_1	4	1272	890	25.08	3014	6028	240
mice_4_2	4	1272	890	25.08	3014	6028	240
zebrafish_010_1	10	3712	3712	32.00	3840	7680	240
zebrafish_010_2	10	3712	3712	32.00	3840	7680	240
zebrafish_010_3	10	3712	3712	32.00	3840	7680	240
zebrafish_010_4	10	1148	946	28.12	3374	6748	240
zebrafish_020	20	1008	1018	29.08	3492	6984	240
zebrafish_060_1	60	3712	3740	32.00	3840	7680	240
zebrafish_080_1	80	3712	3740	32.00	3840	7680	240
zebrafish_100_1	100	3712	3740	32.00	3840	7680	240
zebrafish_100_2	100	3584	3400	32.00	3840	7680	240
zebrafish_100_3	100	3584	3500	32.00	3840	7680	240

Table S2: Dataset characteristics and segmentation diagnostics. For each recording, the table reports the average animal crossing/overlap rate, average trajectory fragmentation (expressed as fragments per animal-minute), segmentation rate observed across perturbation levels, and the maximum segmentation-rate variation induced by visual perturbations (Δ0–Δ2). These metrics were included to assess whether the observed reductions in identity consistency could be explained by substantial changes in segmentation quality. All videos were selected from the datasets released with *new idtracker.ai*.

Dataset	Avg Animal Crossing/Overlap	Avg Fragments (per-animal minute)	Segmentation Rate (min - max)	Segmentation Rate (variation with Δ)
drosophila_010	0.0007	4.91	0.99 - 1.00	1.00 %
drosophila_038	0.0294	3.88	0.95 - 1.00	1.34 %
drosophila_072	0.0010	1.90	1.00 - 1.00	0.21 %
drosophila_080	0.0090	3.42	0.99 - 1.00	0.19 %
drosophila_100_1	0.0115	5.10	1.00 - 1.01	0.65 %
drosophila_100_2	0.0074	4.18	1.00 - 1.01	0.40 %
drosophila_100_3	0.0108	5.53	0.99 - 1.00	0.25 %
mice_2_1	0.1296	17.50	0.90 - 0.90	0.00 %
mice_2_2	0.1316	18.38	0.85 - 0.90	2.78 %
mice_2_3	0.0869	10.00	0.90 - 0.95	0.00 %
mice_2_4	0.0606	10.88	0.95 - 0.95	0.00 %
mice_4_1	0.0532	14.13	0.95 - 0.95	0.00 %
mice_4_2	0.0395	5.91	0.95 - 0.98	0.00 %
zebrafish_010_1	0.0084	7.63	0.99 - 0.99	0.00 %
zebrafish_010_2	0.0163	14.44	0.98 - 0.99	0.00 %
zebrafish_010_3	0.0072	8.04	0.99 - 0.99	0.00 %
zebrafish_010_4	0.0020	2.93	0.99 - 1.00	0.50 %
zebrafish_020	0.0107	3.38	0.99 - 0.99	0.25 %
zebrafish_060_1	0.0060	7.29	0.99 - 1.00	0.08 %
zebrafish_080_1	0.0140	14.44	0.99 - 0.99	0.25 %
zebrafish_100_1	0.0333	21.90	0.97 - 0.98	0.31 %
zebrafish_100_2	0.0076	8.18	0.99 - 0.99	0.00 %
zebrafish_100_3	0.0078	7.90	0.99 - 0.99	0.10 %

Table S3: Inter-session consistency (with retraining). Identity consistency (mean and standard deviation) is reported for each dataset under Δ0, Δ1, and Δ2 visual mismatch levels when retraining is enabled. Consistency is evaluated using the overlapping anchor frame between Session 1 and Session 2.

Dataset	Δ0 (Control)	Δ1 Mean	Δ2 Mean	Δ1 StD	Δ2 StD
drosophila_010	1.00	0.59	0.44	0.27	0.16
drosophila_038	0.76	0.87	0.70	0.02	0.03
drosophila_072	0.97	0.87	0.61	0.12	0.13
drosophila_080	0.95	0.64	0.53	0.20	0.04
drosophila_100_1	0.95	0.61	0.33	0.04	0.06
drosophila_100_2	0.99	0.65	0.34	0.04	0.08
drosophila_100_3	0.98	0.89	0.80	0.03	0.01
mice_2_1	1.00	1.00	1.00	0.00	0.00
mice_2_2	1.00	1.00	1.00	0.00	0.00
mice_2_3	1.00	1.00	1.00	0.00	0.00
mice_2_4	1.00	1.00	1.00	0.00	0.00
mice_4_1	1.00	0.75	0.38	0.35	0.18
mice_4_2	1.00	0.75	0.50	0.35	0.00
zebrafish_010_1	1.00	0.65	0.65	0.49	0.49
zebrafish_010_2	1.00	1.00	1.00	0.00	0.00
zebrafish_010_3	1.00	1.00	0.90	0.00	0.14
zebrafish_010_4	1.00	0.80	0.65	0.28	0.21
zebrafish_020	1.00	0.94	0.48	0.08	0.41
zebrafish_060_1	1.00	0.96	0.51	0.05	0.08
zebrafish_080_1	1.00	0.97	0.36	0.04	0.03
zebrafish_100_1	1.00	0.89	0.24	0.04	0.09
zebrafish_100_2	1.00	0.97	0.19	0.02	0.08
zebrafish_100_3	1.00	0.92	0.34	0.02	0.17
Mean	0.98	0.86	0.61	0.11	0.10
StD	0.05	0.15	0.27	0.14	0.13
Max	1.00	1.00	1.00	0.49	0.49
Min	0.76	0.59	0.19	0.00	0.00

Table S4: Intra-session consistency (with retraining). Identity consistency (mean and standard deviation) is reported for each dataset under Δ0, Δ1, and Δ2 visual mismatch levels when retraining is enabled. Consistency is evaluated using randomly sampled pairs of frames within Session 2.

Dataset	Δ0 (Control)	Δ1 Mean	Δ2 Mean	Δ1 StD	Δ2 StD
drosophila_010	1.00	1.00	0.98	0.00	0.03
drosophila_038	0.95	0.92	0.93	0.05	0.04
drosophila_072	1.00	1.00	1.00	0.00	0.01
drosophila_080	1.00	0.98	0.98	0.02	0.02
drosophila_100_1	0.97	0.96	0.88	0.03	0.04
drosophila_100_2	0.99	0.99	0.98	0.01	0.01
drosophila_100_3	0.99	0.88	0.92	0.08	0.05
mice_2_1	0.80	0.71	0.80	0.41	0.38
mice_2_2	0.82	0.77	0.79	0.39	0.39
mice_2_3	0.93	0.84	0.90	0.31	0.28
mice_2_4	0.99	0.97	0.98	0.14	0.07
mice_4_1	0.97	0.93	0.97	0.16	0.12
mice_4_2	0.99	0.98	1.00	0.09	0.04
zebrafish_010_1	1.00	1.00	0.99	0.02	0.02
zebrafish_010_2	1.00	1.00	0.99	0.02	0.02
zebrafish_010_3	1.00	1.00	0.98	0.02	0.03
zebrafish_010_4	1.00	1.00	0.99	0.01	0.02
zebrafish_020	1.00	0.99	0.99	0.02	0.01
zebrafish_060_1	1.00	1.00	0.96	0.01	0.02
zebrafish_080_1	1.00	0.99	0.96	0.01	0.02
zebrafish_100_1	1.00	0.99	0.98	0.01	0.01
zebrafish_100_2	1.00	0.99	0.84	0.01	0.03
zebrafish_100_3	1.00	0.99	0.96	0.01	0.01
Mean	0.97	0.95	0.95	0.08	0.07
StD	0.06	0.08	0.06	0.12	0.11
Max	1.00	1.00	1.00	0.41	0.39
Min	0.80	0.71	0.79	0.00	0.01

Table S5: Inter-session consistency (without retraining). Identity consistency (mean and standard deviation) is reported for each dataset under Δ0, Δ1, and Δ2 visual mismatch levels when retraining is disabled. Consistency is evaluated using the overlapping anchor frame between Session 1 and Session 2.

Dataset	Δ0 (Control)	Δ1 Mean	Δ2 Mean	Δ1 StD	Δ2 StD
drosophila_010	1.00	0.64	0.38	0.35	0.18
drosophila_038	0.76	0.83	0.71	0.05	0.00
drosophila_072	0.97	0.68	0.59	0.00	0.12
drosophila_080	0.97	0.55	0.42	0.25	0.04
drosophila_100_1	0.94	0.47	0.22	0.12	0.07
drosophila_100_2	0.99	0.59	0.30	0.04	0.05
drosophila_100_3	0.96	0.88	0.71	0.01	0.02
mice_2_1	1.00	0.50	0.50	0.71	0.71
mice_2_2	1.00	1.00	1.00	0.00	0.00
mice_2_3	1.00	1.00	1.00	0.00	0.00
mice_2_4	1.00	1.00	1.00	0.00	0.00
mice_4_1	1.00	0.75	0.38	0.35	0.18
mice_4_2	1.00	0.83	0.38	0.24	0.18
zebrafish_010_1	1.00	0.55	0.55	0.64	0.64
zebrafish_010_2	1.00	0.90	0.82	0.14	0.02
zebrafish_010_3	1.00	1.00	0.94	0.00	0.09
zebrafish_010_4	1.00	0.80	0.65	0.28	0.21
zebrafish_020	1.00	0.94	0.48	0.08	0.41
zebrafish_060_1	1.00	0.91	0.26	0.08	0.00
zebrafish_080_1	1.00	0.87	0.18	0.14	0.02
zebrafish_100_1	1.00	0.75	0.16	0.01	0.18
zebrafish_100_2	1.00	0.93	0.18	0.01	0.08
zebrafish_100_3	1.00	0.87	0.20	0.03	0.14
Mean	0.98	0.79	0.52	0.15	0.14
StD	0.05	0.17	0.28	0.20	0.19
Max	1.00	1.00	1.00	0.71	0.71
Min	0.76	0.47	0.16	0.00	0.00

Table S6: Intra-session consistency (without retraining). Identity consistency (mean and standard deviation) is reported for each dataset under Δ0, Δ1, and Δ2 visual mismatch levels when retraining is disabled. Consistency is evaluated using randomly sampled pairs of frames within Session 2.

Dataset	Δ0 (Control)	Δ1 Mean	Δ2 Mean	Δ1 StD	Δ2 StD
drosophila_010	1.00	0.88	0.75	0.12	0.12
drosophila_038	0.87	0.75	0.77	0.06	0.10
drosophila_072	0.95	0.92	0.80	0.04	0.07
drosophila_080	0.84	0.70	0.58	0.10	0.10
drosophila_100_1	0.88	0.59	0.32	0.14	0.17
drosophila_100_2	0.99	0.84	0.64	0.04	0.14
drosophila_100_3	0.96	0.86	0.72	0.07	0.09
mice_2_1	0.78	0.42	0.55	0.46	0.47
mice_2_2	0.82	0.77	0.80	0.39	0.39
mice_2_3	0.93	0.84	0.89	0.31	0.30
mice_2_4	0.99	0.60	0.69	0.46	0.46
mice_4_1	0.95	0.57	0.34	0.26	0.31
mice_4_2	0.99	0.70	0.63	0.17	0.25
zebrafish_010_1	1.00	0.65	0.67	0.15	0.15
zebrafish_010_2	1.00	0.95	0.73	0.08	0.20
zebrafish_010_3	1.00	0.81	0.83	0.12	0.11
zebrafish_010_4	1.00	1.00	0.89	0.01	0.07
zebrafish_020	1.00	0.85	0.73	0.09	0.16
zebrafish_060_1	1.00	0.84	0.41	0.07	0.18
zebrafish_080_1	1.00	0.69	0.33	0.09	0.15
zebrafish_100_1	1.00	0.67	0.27	0.10	0.17
zebrafish_100_2	1.00	0.72	0.29	0.08	0.18
zebrafish_100_3	1.00	0.70	0.34	0.12	0.18
Mean	0.95	0.75	0.61	0.15	0.20
StD	0.07	0.14	0.21	0.13	0.11
Max	1.00	1.00	0.89	0.46	0.47
Min	0.78	0.42	0.27	0.01	0.07

Table S7. Summary of intra-session mixed-effects model. Results of the linear mixed-effects model fitted using intra-session identity consistency as the response variable. Perturbation level (Δ), retraining condition, and group size (number of animals) were included as fixed effects, and dataset was included as a random intercept. The table reports the estimated regression coefficients (β), corresponding 95 % confidence intervals (CI), and p-values for the effects included in the model.

Predictor (Intra-session)	Effect	β (Effect Size)	95 % CI	p-value
Δ1	Reduced consistency	-0.112	[-0.162, -0.062]	<0.001
Δ2	Reduced consistency	-0.188	[-0.238, -0.137]	<0.001
Retraining	Improved consistency	0.185	[0.144, 0.226]	<0.001
Group size	No significant	-0.000	[-0.001, 0.000]	0.433
Δ1 x Retraining	Retraining mitigates the negative effect of Δ1	0.179	[0.097, 0.261]	<0.001
Δ2 x Retraining	Retraining mitigates the negative effect of Δ2	0.319	[0.237, 0.401]	<0.001

Table S8. Summary of inter-session mixed-effects model. Results of the linear mixed-effects model fitted using identity consistency as the response variable. Perturbation level (Δ), retraining condition, and group size (number of animals) were included as fixed effects, and dataset was included as a random intercept. The table reports the estimated regression coefficients (β), corresponding 95 % confidence intervals (CI), and p-values for the effects included in the model.

Predictor (Inter-session)	Effect	β (Effect Size)	95 % CI	p-value
Δ1	Reduced consistency	-0.157	[-0.221, -0.094]	<0.001
Δ2	Reduced consistency	-0.418	[-0.481, -0.355]	<0.001
Retraining	No significant	0.050	[-0.002, 0.101]	0.06
Group size	Reduced consistency with larger groups	-0.002	[-0.003, -0.001]	0.001
Δ1 x Retraining	No significant mitigation of Δ1 by retraining	0.064	[-0.063, 0.191]	0.323
Δ2 x Retraining	No significant mitigation of Δ2 by retraining	0.084	[-0.043, 0.211]	0.195

Table S9. Statistical comparisons between within-session and cross-session evaluations. Paired comparisons across the nine rotating session configurations for accuracy and similarity gap. Mean differences, 95% confidence intervals (CI), p-values, and effect sizes (Cohen's dz) are reported. Negative differences indicate lower accuracy or reduced identity separation in cross-session evaluations relative to the corresponding within-session condition. Effect sizes are reported as Cohen's dz, a standardized measure for paired comparisons calculated as the mean paired difference divided by the standard deviation of the paired differences.

Comparison	Metric	Mean difference	95 % CI	p-value	Cohen's dz
Single-session vs Single cross-session	Accuracy	-0.782	[-0.831, -0.733]	<0.001	-16.554
Single-session vs Single cross-session	Similarity gap	-1.037	[-1.105, -0.968]	<0.001	-15.262
Multi-session vs Multi cross-session	Accuracy	-0.491	[-0.538, -0.444]	<0.001	-11.008
Multi-session vs Multi cross-session	Similarity gap	-0.588	[-0.648, -0.528]	<0.001	-9.946
